# RETRACTION: Differential Involvement of Ras‐GRF1 and Ras‐GRF2 in L‐DOPA‐Induced Dyskinesia

**DOI:** 10.1002/acn3.70515

**Published:** 2026-08-20

## Abstract

**RETRACTION**: S. Bido, N. Solari, M. Indrigo, A. D’Antoni, R. Brambilla, M. Morari, and S. Fasano, “Differential Involvement of Ras‐GRF1 and Ras‐GRF2 in L‐DOPA‐Induced Dyskinesia,” *Annals of Clinical and Translational Neurology* 2, no. 6 (2015): 662–678, https://doi.org/10.1002/acn3.202.

The above article, published online on 24 April 2015 in Wiley Online Library (wileyonlinelibrary.com) has been retracted by agreement between the journal Editor‐in‐Chief, Ahmet Hoke; American Neurological Association; and John Wiley & Sons, Inc. US. The retraction has been agreed upon following an investigation into concerns raised by a third party. Several image overlaps and duplicated elements in Figures 1, 3, 6, and 7 were identified across different panels even though the corresponding images represent different samples. Accordingly, the editors have lost confidence in the data presented and consider the conclusions of this manuscript insufficiently supported. The authors have been informed of the decision to retract but remained unresponsive.

**RETRACTION**: S. Bido, N. Solari, M. Indrigo, A. D’Antoni, R. Brambilla, M. Morari, and S. Fasano, “Differential Involvement of Ras‐GRF1 and Ras‐GRF2 in L‐DOPA‐Induced Dyskinesia,” *Annals of Clinical and Translational Neurology* 2, no. 6 (2015): 662–678, https://doi.org/10.1002/acn3.202.

The above article, published online on 24 April 2015 in Wiley Online Library (wileyonlinelibrary.com) has been retracted by agreement between the journal Editor‐in‐Chief, Ahmet Hoke; American Neurological Association; and John Wiley & Sons, Inc. US. The retraction has been agreed upon following an investigation into concerns raised by a third party. Several image overlaps and duplicated elements in Figures 1, 3, 6, and 7 were identified across different panels even though the corresponding images represent different samples. Accordingly, the editors have lost confidence in the data presented and consider the conclusions of this manuscript insufficiently supported. The authors have been informed of the decision to retract but remained unresponsive.

